# New Potent 5α- Reductase and Aromatase Inhibitors Derived from 1,2,3-Triazole Derivative

**DOI:** 10.3390/molecules25030672

**Published:** 2020-02-05

**Authors:** Mohamed El-Naggar, Amira S. Abd El-All, Shweekar I. A. El-Naem, Mohamed M. Abdalla, Huda R. M. Rashdan

**Affiliations:** 1 Chemistry Department, Faculty of Sciences, University of Sharjah, Sharjah 27272, UAE; melnagrr@sharjah.ac.ae; 2 Chemistry of Natural and Microbial Products Department, Pharmaceutical and Drug Industries Research Division, National Research Centre, Dokki, Cairo 12622, Egypt; amira@yahoo.com (A.S.A.E.-A.); shwekar@yahoo.com (S.I.A.E.-N.); 3 Research and Development Unit, Saco Pharm. Co., 6 October City 11632, Egypt; Abdallah@gmail.com

**Keywords:** 1,2,3-triazoles, 5-α reductase inhibitors, aromatase inhibitors, hormone-dependent cancer

## Abstract

This work describes the utility of pyrazole-4-carbaldehyde **1** as starting material for the synthesis of a novel potent series of 5α-reductase and aromatase inhibitors derived from 1,2,3-triazole derivative. Condensation of **1** with active methylene and different amino pyrazoles produced the respective Schiff bases **2**–**4**, **8** and **9**. On the other hand, **1** was reacted with ethyl cyanoacetate and thiourea in one-pot reaction to afford the pyrazolo-6- thioxopyridin-2-[3*H*]-one (**10**). Moreover, α–β unsaturated chalcone derivative **11** was prepared via the reaction of compound **1** with *P*-methoxy acetophenone, which in turn reacted with each of ethyl cyanoacetate, malononitrile, hydrazine hydrate, and thiosemicarbazide to afford the corresponding pyridine and pyrazole derivatives **13**, **14**, **17,** and **20.** The structure of newly synthesized compounds was characterized by analytical and spectroscopic data (IR, MS and NMR). All new compounds were evaluated against 5α-reductase and aromatase inhibitors and the results showed that many of these compounds inhibit 5α-reductase and aromatase activity; compound **13** was found to be the highest potency among the tested samples comparing with the reference drugs.

## 1. Introduction

The pharmaceutical importance of 1,2,3-triazole ring is due to its efficacy as c-Met kinase inhibitors [1], aromatase inhibitors [2], 5α-reductase inhibitors [3], virostatic [4], antiproliferative agents [5,6,7], GABA-antagonists [8], antimicrobial agents [9,10], anti-bacterial agents [11], novel inhibitors of human immunodeficiency virus type1 protease [12], glycosidase inhibitors, antimalarial agents [13], antihistaminic agents [14], nucleosides [15], as synthetic intermediates for some antibiotic compounds [16], and also for treatment of Alzheimer’s disease [17].

On the other hand, pyrazoline derivatives are a very important class of heterocyclic compounds characterized by biological activities as antiviral [18], anticancer [19], antimicrobial agent [20], 5α-reductase, and aromatase inhibitors [21,22].

We are incorporated in a research group aimed to produce biologically active heterocyclic compounds using available chemicals [5,6,23,24,25,26,27,28,29,30,31]. Our growing interest in this manuscript is incorporation between 1,2,3-triazole ring and pyrazoline ring in a new heterocyclic compound **1** [5]. The starting compound **1** will react with appropriate active methylene to give compounds **2–4**, while, a newly substituted pyrazole derivative **5–9** will be formed when compounds **2–4** react with hydrazine hydrate and different amino pyrazole. Chalcone derivative **11** is prepared via the reaction substituted pyrazole **1** with *P*-methoxy acetophenone, which in turn reacts with each of ethyl cyanoacetate, malononitrile hydrazine hydrate, and thiosemicarbazide to give the corresponding pyridine and pyrazole derivatives **13**, **14**, **17,** and **20**, respectively. The newly synthesized compounds were evaluated against 5-α reductase and aromatase inhibitors to study their activities as anti- hormone-dependent cancer compounds.

## 2. Results and Discussion

### 2.1. Chemistry

It has been found that the reaction of pyrazole (3-(5-methyl-1-phenyl-1*H*-1,2,3-triazol-4-yl)-1-phenyl-1*H*-pyrazole-4-carbaldehyde (**1**) with some selected active methylene (namely, malononitrile, acetyl acetone and diethyl malonate) with the presence of triethylamine in ethanol gave the respective new 3-(5-methyl-1-phenyl-1*H*-1,2,3-triazol-4-yl)-1-phenyl-1*H*-pyrazol-4-yl)methylene) derivatives **2–4**. The structures of compounds **2–4** were confirmed on the basis of their microanalytical and spectral data. For compound 2-((3-(5-methyl-1-phenyl-1*H*-1,2,3-triazol-4-yl)-1-phenyl-1*H*-pyrazol-4-yl)methylene) malononitrile (**2**), taken as a representative example, its IR spectrum (KBr/cm^−1^) revealed a strong sharp absorption band at *v* 2222 cm^−1^ corresponding to C≡N group and two absorption bands at *v* 1620 cm^−1^ and 1640 cm^−1^due to C=C and C=N. Its ^1^H-NMR spectrum (DMSO-*d_6_*, *δ* ppm.) showed three signals at *δ* = 2.52, 6.37 and 8.34 ppm. due to -CH_3_ and -CH pyrazole and CH-olefinic of Schiff base protons, respectively. The aromatic protons (10H) appeared as a multiplet at *δ* = 7.33–7.94 ppm. Moreover, the structure was also supported by its mass spectrum (*m/z* 378) [M^+^], which agrees with its molecular formula C_22_H_15_N_7_.

**Scheme 1:**
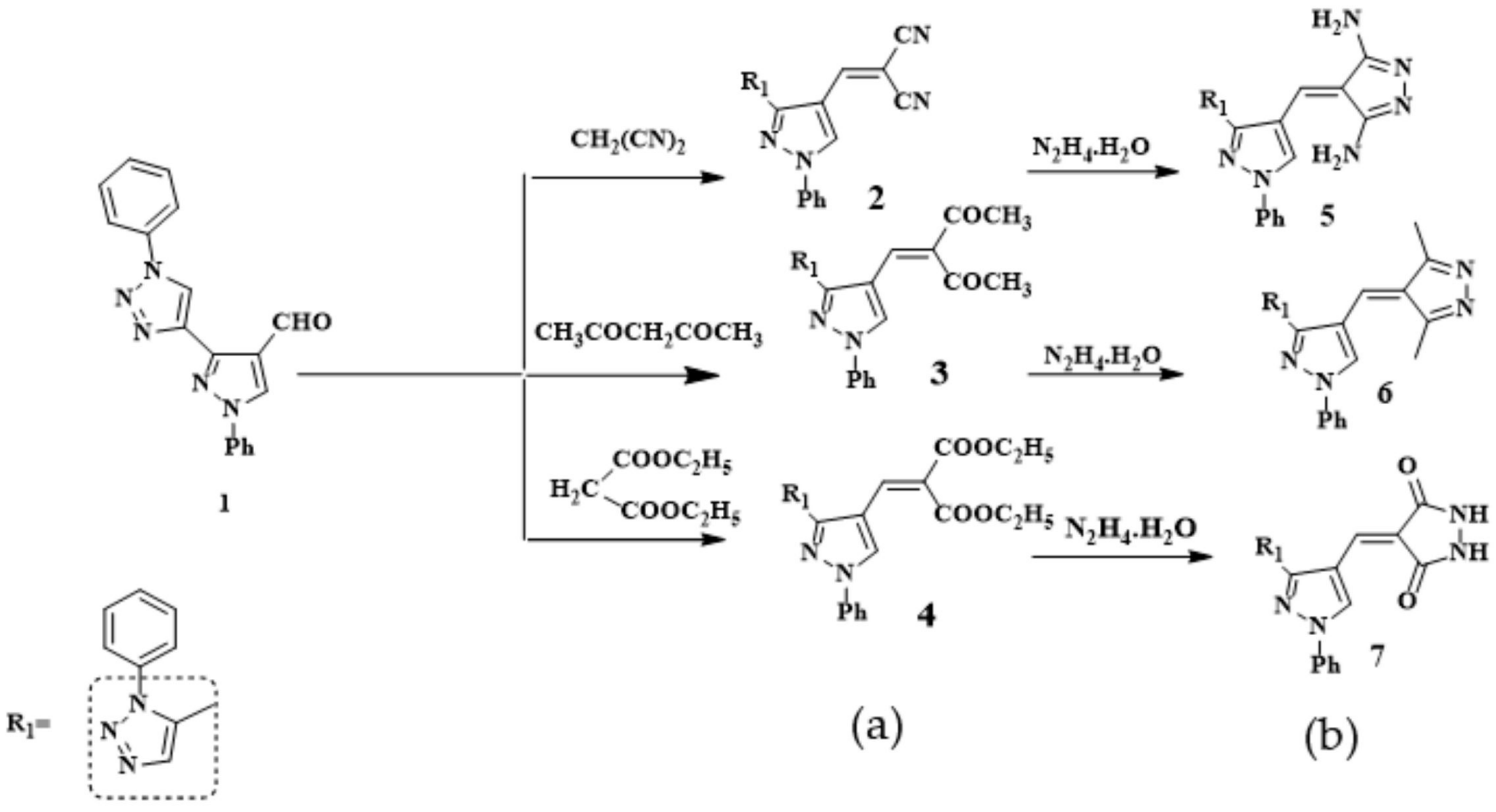
Reaction of compound 1 with (**a**) active methylenes; (**b**) the products with hydrazine hydrate.

The newly synthesized Schiff bases derivatives **2**–**4** have now been investigated as key molecules for building new fused pyrazole derivatives through the condensation reaction on their carboxylic double bond with nucleophile hydrazine hydrate, to give compounds **5**–**7** respectively (Scheme 1). The structures of **5**–**7** were established on the basis of their elemental analysis and spectral data (MS, IR, and ^1^H-NMR). As an example, structure **6** was supported by its mass spectrum (*m/z* 407) [M^+^], which agrees with its molecular formula C_24_H_21_N_7_. Its IR spectrum (KBr/cm^−1^) showed an absorption band at *v* 1600 cm^−1^, which corresponds to the C=C, a band at *v* 1620 cm^−1^ due to C=N, and a band at *v* 2862, 2924 cm^−1^ue to CH-aliphatic. Its ^1^H-NMR spectrum displayed four singlet signals at *δ* = 2.53, 2.75, 6.37, and 8.35 ppm. due to two -CH_3_, CH-pyrazole and CH-olefinic, respectively, one multiple signal at δ = 7.35–7.84 ppm. related to the 10 protons of aromatic. he other bands of compounds **5** and **7** appeared in IR, ^1^H-NMR spectra and molecular weight determination (MS) in the expected regions.

**Scheme 2:**
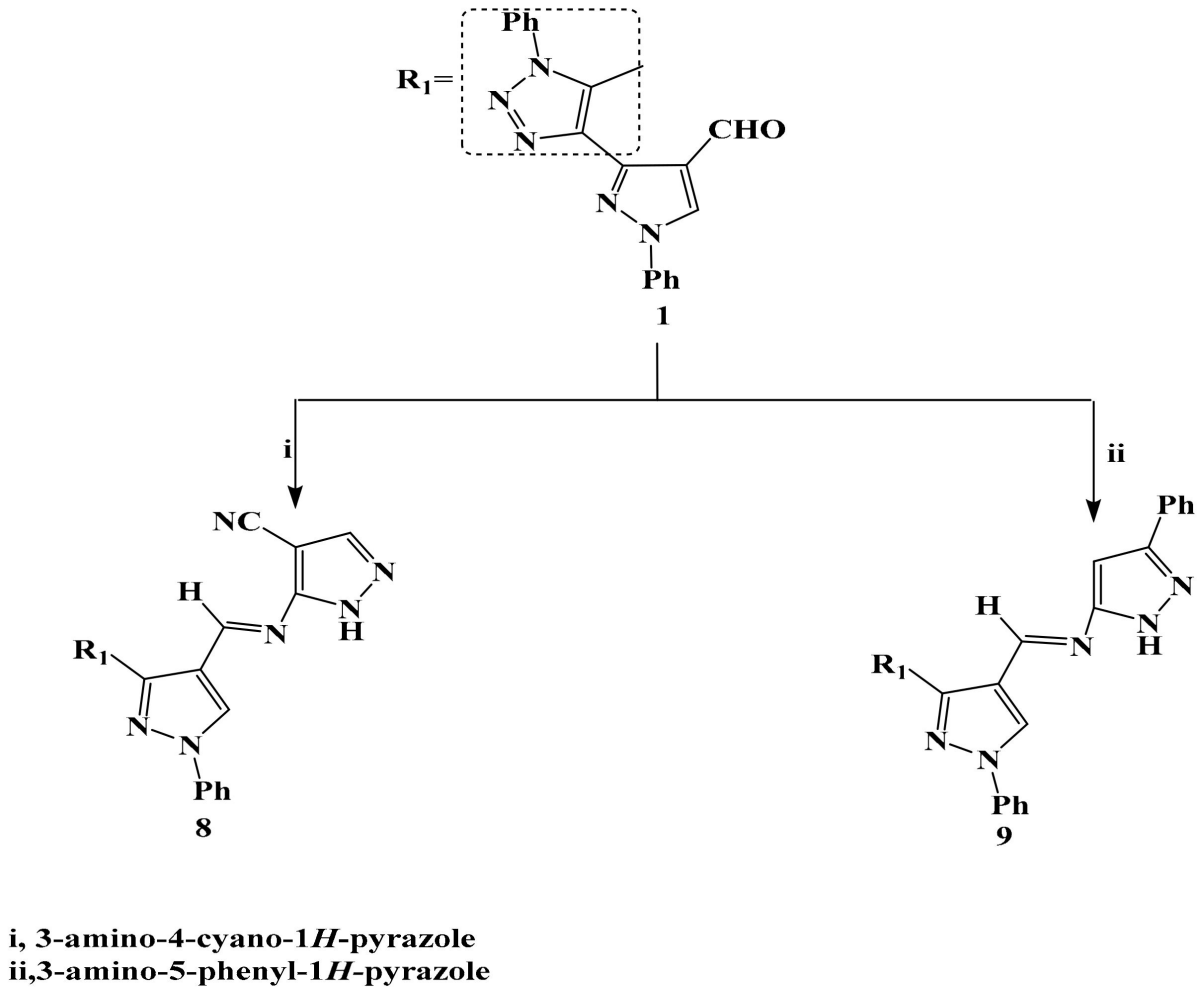
The reaction of compound 1 with amino pyrazoles.

Compound **1** was allowed to react with each of 4-cyano-3-aminopyrazole and 5-phenyl-3-aminopyrazole in boiling glacial acetic acid via Schiff-base to afford the corresponding **8** and **9** respectively in a good yield (Scheme 2). The structure of compound **8** and **9** were confirmed by different spectroscopic tools; structure **8** was supported by its mass spectrum (*m/z* 419) [M^+^], which agrees with its molecular formula C_23_H_17_N_9_. Its IR spectrum (KBr/cm^−1^) revealed a strong absorption band at *v* 2222 cm^−1^ due to C≡N and a strong absorption band at *v* 3105 cm^−1^ attributed to the NH group. ^1^H-NMR spectrum of compounds **8** and **9** revealed a proton at δ = 8.50 and 8.51 ppm. which was assigned to the appearance of the CH-olefinic of Schiff-base. The other bands of compounds **8** and **9** appeared in IR, ^1^H-NMR spectra and molecular weight determination (MS) in the expected regions.

**Scheme 3:**
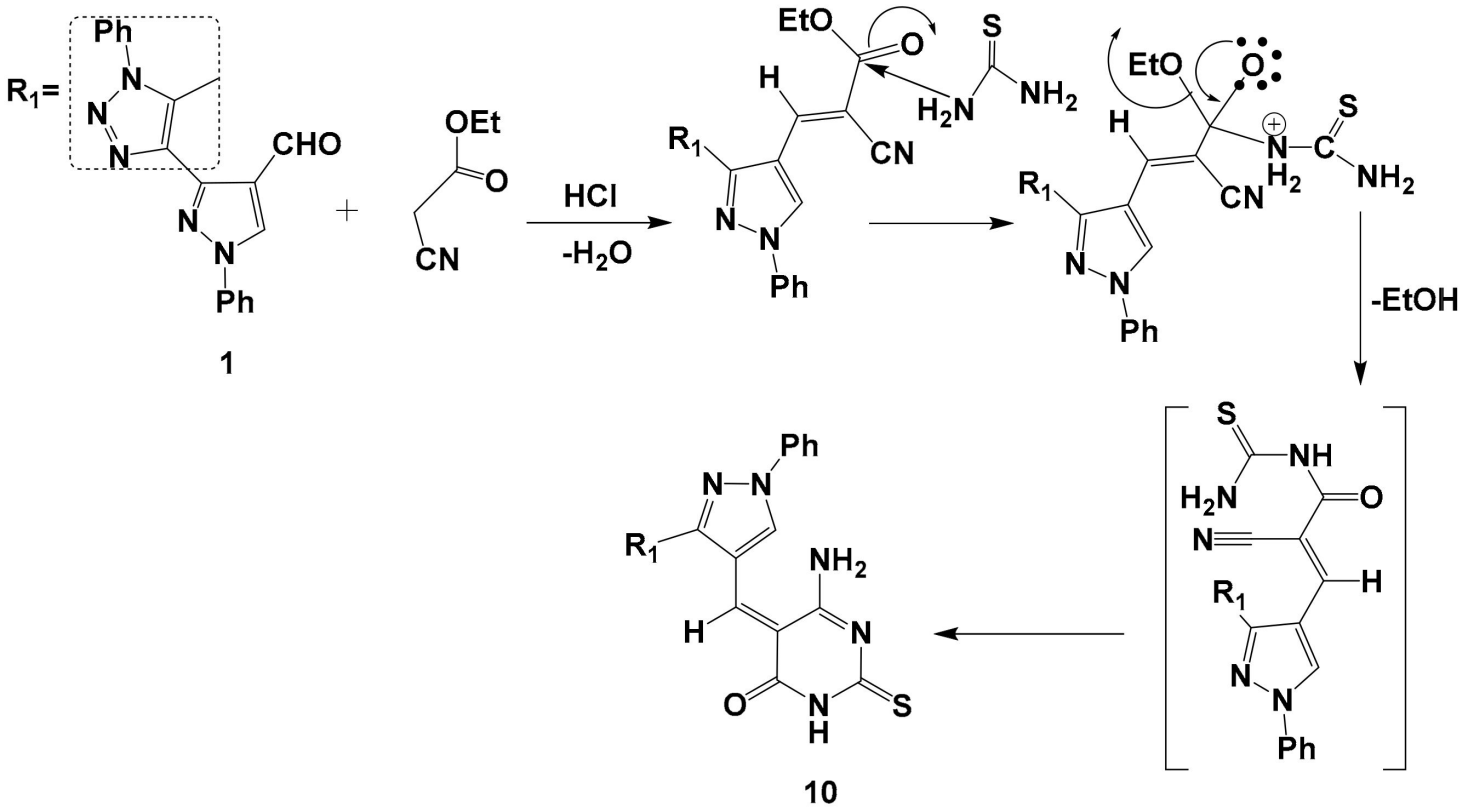
The suggested mechanism for preparation of compound **10**.

It has now been found that one-pot reaction of compound **1** with ethyl cyanoacetate and thiourea in the presence of hydrochloric acid in refluxing ethanol afforded the corresponding derivative **10** through the mechanism illustrated in (Scheme 3). The reaction proceeded via tetrahedral mechanism, in which the N-C bond was formed before the CO bond started to break and ethanol eliminated, and consequently a lot of energy was accumulated in the reaction medium, which offset the activation energy of the reaction and a facile conversion occurred; then cyclization took place via the addition of an amino group to the nitrile group to yield the desired product **10**. Structure **10** was supported by its mass spectrum (*m/z* 454) [M^+^], which agrees with its molecular formula C_23_H_18_N_8_OS. Its IR spectrum (KBr/cm^−1^) showed a strong absorption band at *v* 1400 cm^−1^, which is attributed to C=S, a strong absorption band at *v* 3228 cm^−1^ due to (NH) and a strong absorption forked band at *v* 3317, 3348 cm^−1^due to the NH_2_ group. Its ^1^H-NMR spectrum displayed five singlets at *δ* = 2.53, 6.31, 6.82, 8.21, and 9.50 ppm. due to CH_3_, CH-pyrazole, NH_2_group, CH-olefinic, and NH protons, respectively, and one multiple at *δ* = 7.35-7.84 ppm. related to the 10 protons of aromatic.

**Scheme 4:**
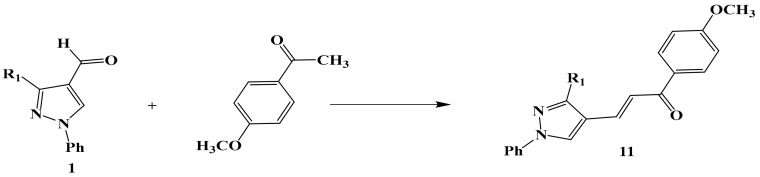
The reaction of compound 1 with *p*-methoxy acetophenone to form chalcone **11**.

Otherwise, compound number **1** reacted with *P*-methoxy acetophenone to give the α–*β* unsaturated derivative 1-(4-methoxyphenyl)-3-(3-(5-methyl-1-phenyl-1*H*-1,2,3-triazol-4-yl)-1-phenyl-1*H*-pyrazol-4-yl)prop-2-en-1-one **11** (Scheme 4).

**Scheme 5:**
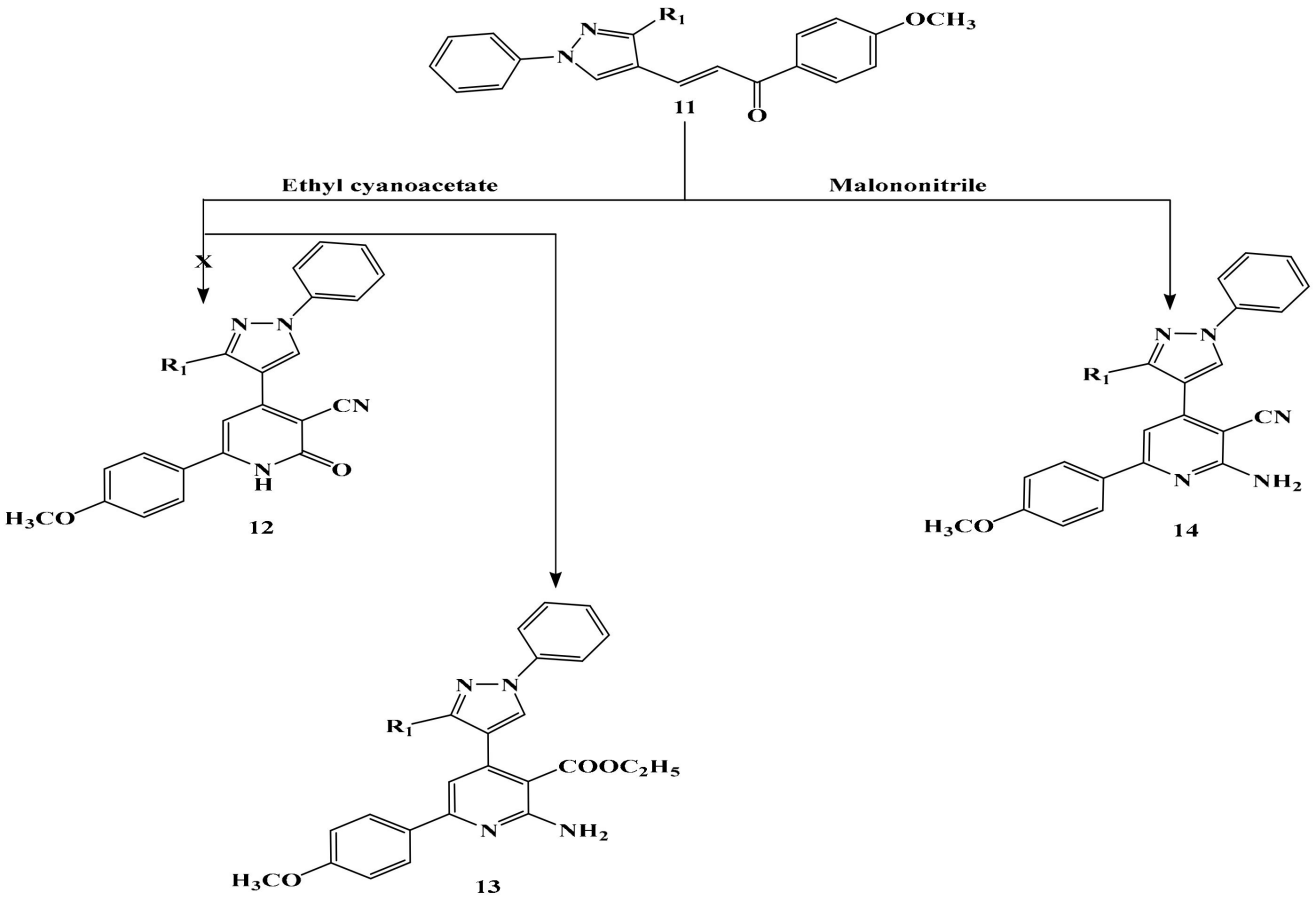
The reaction of chalcone **11** with ethyl cyanoacetate and malononitrile.

The structure of chalcone **11** was inferred from spectral data (IR, Mass, NMR), elemental analysis and chemical transformation. However, the structure of **11** was inferred chemically via its interaction with each of ethyl cyanoacetate and malononitrile to give the corresponding **13** and **14** respectively (Scheme 5). The structure of compound **13** and **14** was confirmed by spectral data (IR, Mass, NMR) and correct elemental analysis. The IR spectrum of compound **13** showed a strong absorption band at *v* 3066, 3133 cm^−1^ attributed to the NH_2_ group, and a strong absorption band at *v* 1740 cm^−1^attributed to the carbonyl ester and devoid of any band for the C≡N group. Its ^1^H-NMR exhibited a triple signal at *δ* = 1.02–1.21 ppm attributed to the three protons of the CH_3_ group of ethyl ester and as a quartet signal *δ* at 3.90–4.12 ppm attributed to the two protons of the CH_2_ group of ethyl ester, which confirmed the formation of compounds **13** and not **12**.

On the other hand, the IR spectrum of compound **14** showed a strong sharp absorption band at 2212 cm^−1^ represented the C≡N which indicated the formation of compound **14**. he other bands of compounds **13** and **14** appeared in IR, ^1^H-NMR spectra molecular weight determination (MS) in the expected regions.

**Scheme 6:**
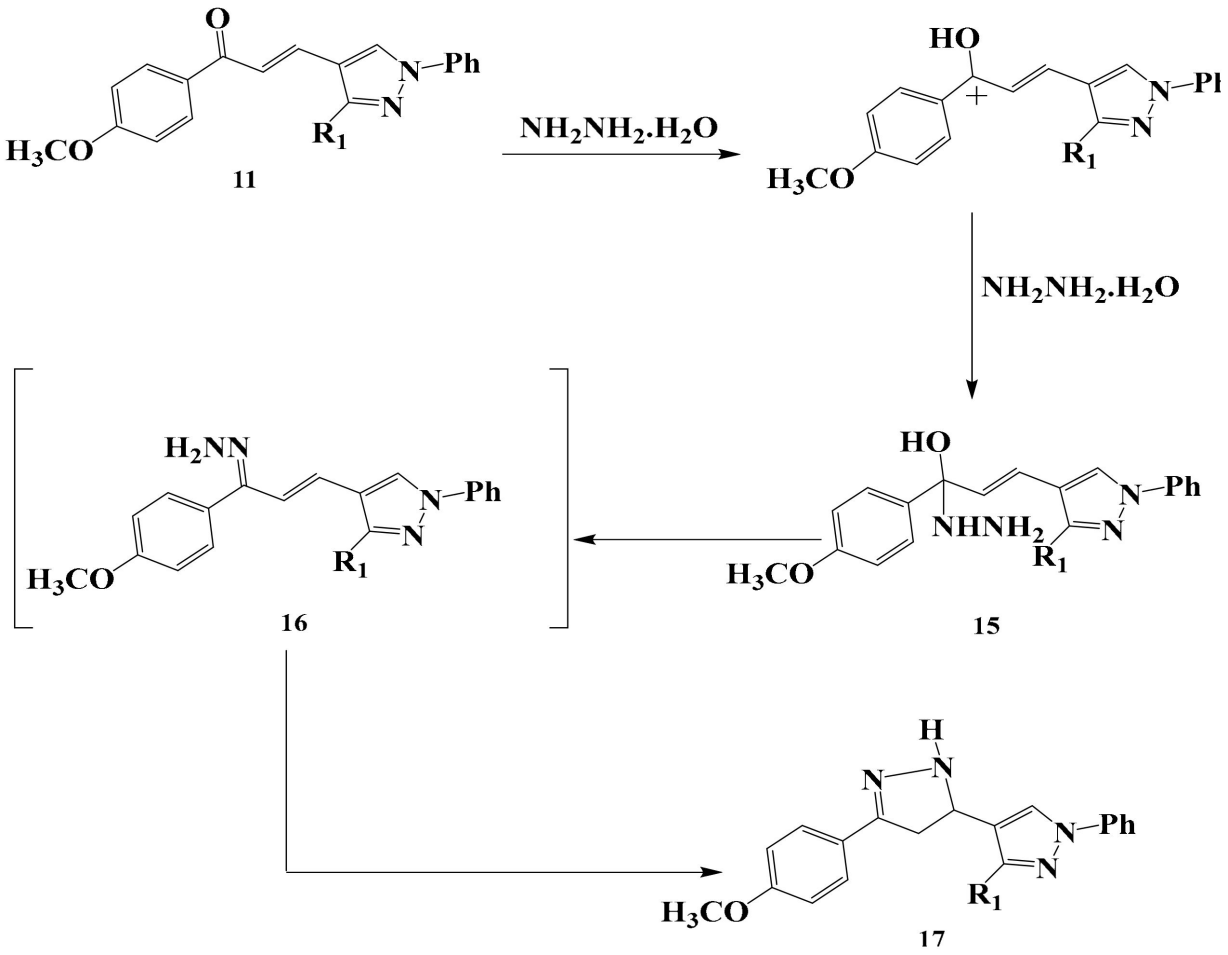
The reaction mechanism of chalcone **11** with hydrazine hydrate to form pyrazole derivative **17**.

When the chalcone **11** was allowed to react with hydrazine hydrate it yielded the corresponding substituted pyrazole **17** in a good yield through the reasonable mechanism discussed in (Scheme 6). The reaction was based through hydrazone derivative followed by ring closure. The structure of compound **17** was confirmed by spectral data (IR, Mass, NMR), elemental analysis and chemical transformation. The bands of compounds **17** appeared in IR, ^1^H-NMR spectra molecular weight determination (MS) in the expected regions.

**Scheme 7:**
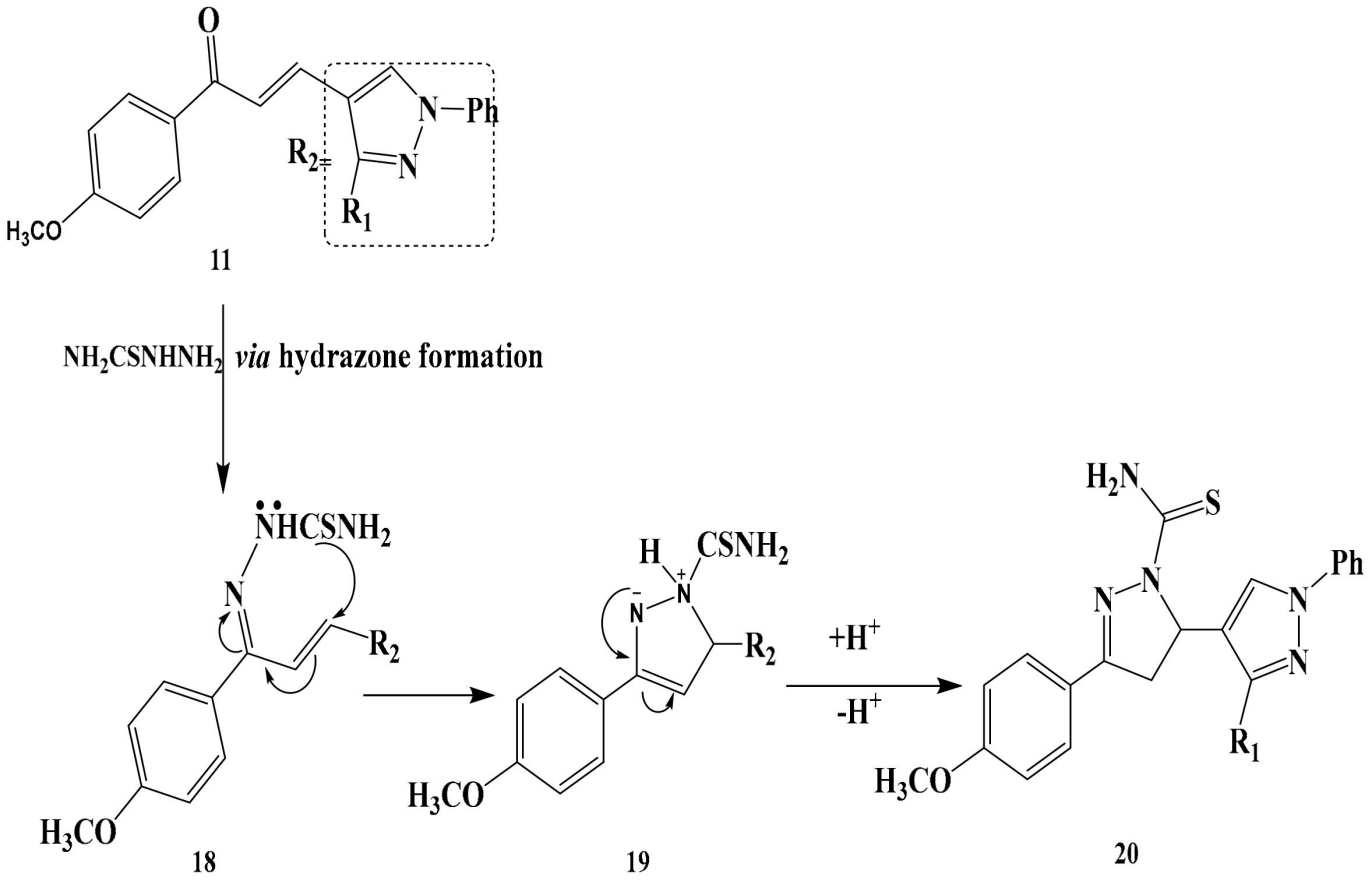
The reaction mechanism of chalcone **11** with thiosemicarbazid to form pyrazoline derivative **20**.

Finally, condensation of chalcone **11** with thiosemicarbazide gave pyrazoline derivative **20** as shown in Scheme 7. The selectivity that leads to the formation of intermediate **18** results from 1,2-addition of thiosemicarbazide to the carbonyl group of chalcone **11** and subsequent N-H intermolecular cycloaddition to the double bond of **18,** which leads to the formation of **20**. As a result and in accordance with the currently accepted mechanism [32], the formation of **20** is formed via hydrazone formation under the reaction condition; the product structure is determined via steps **19–20** where a regioselective enamine-imine tautomerism [33] may take place to form the more stable 2-pyrazoline **20**. The structures of **20** were established on the basis of their elemental analysis and spectral data (MS, IR, ^1^H-NMR, and ^13^C-NMR). Structure **20** was supported by its mass spectrum (*m/z* 454) [M^+^], which agrees with its molecular formula C_23_H_18_N_8_OS. Its IR spectrum (KBr/cm^−1^) showed an absorption band at *v* 3475, 3436 cm^−1^due to NH_2_*,* a band at *v* 2924, 2890 cm^−1^ due to CH-alphatic, a band at *v* 1562 cm^−1^ due to C=C and a due to *v*1616 cm^−1^ due to C=N Its ^1^H-NMR spectrum displayed three singlet signals at *δ* = 2.54, 3.11 and 4.61 ppm due to CH_3_, OCH_3_-NH_2_ group protons respectively, one doublet at *δ* = 3.40-3. 47 related to CH_2_-pyrazole, and two multiples at *δ* = 3.74–3.82 and 7.01–7.72 ppm. related to CH_2_ protons and 15 protons of aromatic.

### 2.2. Pharmacological Screening

#### 2.2.1. α-Reductase Inhibitors

Circulating testosterone and dihydrotestosterone hormone levels or tissue concentrations were measured after the administration of 5α-reductase inhibitor radioimmuno assays. All new synthesized compounds **1**–**11**, **13**, **14**, **17,** and **20** were screened for their 5α-reductase inhibitor activity in vivo. All the tested compounds showed 5α-reductase inhibitor activities with good *IC_50_* (µM); compounds (**4**–**11**, **13**, **14**, **17** and **20**) indicated a significant activity compared to the control drug anastrozole. IC_50_ values were determined. From the results, it is evident that 12 of the investigated compounds have displayed a potent activity as a 5α-Reductase inhibitor (Table 1); the potency of the investigated compounds decreases in the order **13** > **8** > **11** > **20** > **17** > **14** > **7** > **4** > **10** > **6** = **9** > **5**. Compound **13** was found to be the most potent 5α-Reductase inhibitor compared to the other tested compounds, and the standard drug anastrozole may be due to the presence of the ester group and pyridine ring. Compound **8** showed high activity may be due to the presence of the cyano group; also compound **11** giving high activity may be attributed to the presence of α–*β* unsaturated ketone; compound **20** giving high reactivity may be due to the thioamide group; compound **17** giving high activity may be due to the pyrazoline ring system; and compound **14** may be giving high activity due to the cyano-pyridine ring system. Compound **7** giving high activity may be due to the presence of pyrazole and pyrazolone ring systems; compound **4** showing high reactivity may be due to the presence of the pyrazole ring system and ester group; compound **10** giving high reactivity may be due to the presence of the thioprymidone ring system; the activity of compound **6** may be due to the presence of pyrazole ring system and methyl group. Also, the reactivity of compound **9** may be due to the presence of the pyrazole ring system. Finally compound **5** give inhibition activity may be due to the presence of two amino groups, also all the tested compounds have the 1,2,3-triazole moiety, which made them close in structure to that of the standard drug anastrozole. This is all in addition to their physical properties (low melting point, high solubility and small molecular weight), which may increase their reactivity either.

#### 2.2.2. Aromatase Activity Assay

All synthesized compounds **1**–**11**, **13**, **14**, **17,** and **20** were tested for their aromatase inhibitor activity in vivo. From the results given in Table 2, it was found that nine of the investigated compounds (**7**–**11**, **13**, **14**, **17,** and **20**) have given a potent activity as aromatase inhibitors compared to the standard drug letrozole. Compound **13** was found to be the most potent aromatase inhibitor compared to the tested compounds, and the standard drug Letrazole may be due to the presence of the ester group and pyridine ring. Compound **8** showed high activity, which may be due to the presence of the cyano group; also, compound **11** showing high activity may be due to the presence of α-*β* unsaturated ketone; compound **20** showing high reactivity may be due to the thioamide group; and compound **17** showing high activity may be due to the pyrazoline ring system. In addition, compound **14** may have showed high activity due to the cyano-pyridine ring system. Compound **7** showing high activity may be due to the presence of pyrazole and pyrazolone ring systems, and compound **10** showing high reactivity may be due to the presence of the thioprymidone ring system. Also, the reactivity of compound **9** may be due to the presence of the pyrazole ring system. This is all in addition to their physical properties (low melting point, high solubility and small molecular weight), which may increase their reactivity.

## 3. Experimental

### 3.1. General Procedures

All melting points were determined on an electrothermal apparatus and are uncorrected. IR spectra were recorded (KBr discs) on a Shimadzu FT-IR 8201 PC spectrophotometer. H^1^-NMR spectra were recorded in CDCl_3_ and (CD_3_)_2_SO solutions on a Varian Gemini 400 MHz spectrometer, and chemical shifts are expressed in δ ppm units using TMS as an internal reference. Mass spectra were recorded on a GC-MS QP1000 EX Shimadzu. Elemental analyses were carried out at the Microanalytical Center of Cairo University.

### 3.2. Synthesis of Compounds **2**–**4**

#### General Producer

A mixture of **1** [27] (3.3 gm, 10 mmol) and the appropriate amount of malononitrile, acetylacetone and diethylmalonate (10 mmol) was heated under reflux in ethanol (20 mL) containing a catalytic amount of triethyamine (5–10 drops) for 3 h. The reaction mixture was cooled, and the solid formed was separated and recrystallized from the proper solvent to give the corresponding derivatives **2–4**, respectively, in a good yield.

2-((3-(5-Methyl-1-phenyl-1H-1,2,3-triazol-4-yl)-1-phenyl-1H-pyrazol-4-yl)methylene)malononitrile (**2**): Yellow crystals from acetic acid in yield 93%, m.p. 200–202 °C. H^1^-NMR (400 MHz, DMSO-d_6_): δ = 2.52 (s, 3H, CH_3_), 6.37 (s, 1H, CH-pyrazole), 8.34 (s, 1H, CH-aliphatic), 7.33–7.94 (m, 10H, Ar-H). IR (KBr, cm^−1^): v 1620 (C=C), 1640 (C=N), 2222 *v* (CN), 2854, 2927 (CH-aliphatic). MS *m*/*z* (M^+^ 377,54%), (M+1, 378, 26%), (M+2, 379, 5%). Anal. Calcd. for C_22_H_15_N_7_ (377): C, 70.01; H, 4.01; N, 25.98; Found: C, 70.09; H, 4.08; N, 25.90%.

3-((3-(5-Methyl-1-phenyl-1H-1,2,3-triazol-4-yl)-1-phenyl-1H-pyrazol-4-yl)methylene)pentane-2,4-dione (**3**): Pale yellow crystals from acetic acid in yield 81%, m.p. 222–224 °C. H^1^-NMR (400 MHz, DMSO-d_6_): δ = 2.39 (s, 6H, 2CH_3_), 2.52 (s, 3H, CH_3_), 6.33 (s, 1H, CH-pyrazole), 8.34 (s, 1H, CH-aliphatic), 7.37–7.94 (m, 10H, Ar-H). IR (KBr, cm^−1^): *v* 1620 (C=C), 1639 (C=N), 1693 (C=O), 2927, 2974 (CH-aliphatic); MS *m*/*z* (M^+^ 411, 18%), (M+1, 412, 21%), (M+2, 413, 3%). Anal. Calcd. for C_24_H_21_N_5_O_2_ (411): C, 70.06; H, 5.14; N, 17.02; Found: C, 70.01; H, 5.10; N, 17.09%.

Diethyl2-((3-(5-methyl-1-phenyl-1H-1,2,3-triazol-4-yl)-1-phenyl-1H-pyrazol-4-yl)methylene) malonate (**4**): White crystals from ethanol in yield 87%, m.p. 210–212 °C. H^1^-NMR (400 MHz, DMSO-d_6_): δ = 1.24–129 (t, 6H, 2CH_2_CH_3_), 2.53(s, 3H, CH_3_), 4.16–4.23 (q, 4H, 2CH_2_CH_3_), 6.38 (s, 1H, CH-pyrazole), 8.33 (s, 1H, CH-aliphatic), 7.35–7.84 (m, 10H, Ar-H). IR (KBr, cm^−1^): *v* 1620 (C=C), 1632 (C=N), 1697 (C=O), 2862, 2927 (CH-aliphatic). MS: *m*/*z* (M^+^, 471, 23%), (M+1, 472, 6%), (M^+2^ 473, 1%). Anal. Calcd. for C_26_H_25_N_5_O_4_ (471): C, 66.23; H, 5.34; N, 14.85; Found: C, 66.14; H, 5.25; N, 14.89%.

### 3.3. Synthesis of Compounds **5**–**7**

#### General Producer

A mixture of the appropriate of compounds **2**, **3** and **4** (5 mmol) and hydrazine hydrate (1 gm, 1 mL, 10 mmol) was heated under reflux in absolute ethanol (20 mL) for (3 h); the reaction mixture was cooled, and the solid formed was collected and recrystallized to afford the corresponding derivatives **5**, **6** and **7**, respectively.

4-((3-(5-methyl-1-phenyl-1H-1,2,3-triazol-4-yl)-1-phenyl-1H-pyrazol-4-yl)methylene)-4H-pyrazole-3,5-diamine (**5**): White crystals from ethanol in yield 78%, m.p. 240–242 °C. H^1^-NMR (400 MHz, DMSO-d_6_): δ = 2.53 (s, 3H, CH_3_), 5.70 (s, 4H, 2NH_2_), 6.39 (s, 1H, CH-pyrazole), 8.33 (s, 1H, CH-aliphatic), 7.35–7.84 (m, 10H, Ar-H). IR (KBr, cm^−1^): v 1608 (C=C), 1626 (C=N), 2862, 2927 (CH-aliphatic), 3310, 3244 (NH_2_); MS: *m*/*z* (M^+^ 409, 3%), (M+1, 410, 61%), (M+2, 411, 12%). Anal. Calcd. for C_22_H_19_N_9_ (409): C, 64.53; H, 4.68; N, 30.79; Found: C, 64.38; H, 4.55; N, 30.65%.

5-methyl-4-(4-((3,5-dimethyl-4H-pyrazol-4-ylidene)methyl)-1-phenyl-1H-pyrazol-3-yl)-1-phenyl-1H-1,2,3-triazole (**6**): White crystals from ethanol in yield 87%, m.p. 261–263 °C. H^1^-NMR (400 MHz, DMSO-d_6_): δ = 2.53 (s, 3H, CH_3_), 2.75 (s, 6H, 2CH_3_), 6.37 (s, 1H, CH-pyrazole), 8.35 (s, 1H, CH-aliphatic), 7.35–7.84 (m, 10H, Ar-H). IR (KBr, cm^−1^): *v* 1600 (C=C), 1620 (C=N), 2862, 2924 (CH-aliphatic) MS: *m*/*z* (M^+^ 407, 65%), (M^+1^ 408, 12%), (M^+2^ 409, 11%). Anal. Calcd. for C_24_H_21_N_7_ (407): C, 70.74; H, 5.19; N, 24.06; Found: C, 70.55; H, 5.11; N, 23.95%.

4-((3-(5-methyl-1-phenyl-1H-1,2,3-triazol-4-yl)-1-phenyl-1H-pyrazol-4-yl)methylene) pyrazolidine-3,5-dione (**7**): Grey crystals from ethanol in yield 82%, m.p. 282–284 °C. H^1^-NMR (400MHz, DMSO-d_6_): δ = 2.53 (s, 3H, CH_3_), 6.67 (s, 1H, CH-pyrazole), 8.35 (s, 1H, CH-aliphatic), 7.35–7.84 (m, 10H, Ar-H), 11.00 (s, 2H, 2NH). IR (KBr, cm^−1^): *v* 1600 (C=C), 1620 (C=N), 1640 (C=O), 2862, 2924 (CH-aliphatic), 3236 (NH). MS: *m*/*z* (M^+^, 411, 51%), (M+1, 412, 1.2%), (M+2, 413, 17%). Anal. Calcd. for C_22_H_17_N_7_O_2_ (411): C, 64.23; H, 4.16; N, 23.83; Found: C, 64.32; H, 4.09; N, 23.92%.

### 3.4. Synthesis of Compounds **8** and **9**

#### General Producer

A mixture of compound number **1** (3.29 gm, 10 mmol) and the appropriate amount of 3-amino-4-cyano-1*H*-pyrazole and 3-amino-5-phenyl-1*H*-pyrazole was heated under reflux in acetic acid for 2 h. The solid formed through heating was separated and recrystallized from the appropriate solvent to give the corresponding compounds **8** and **9**, respectively.

5-((3-(5-methyl-1-phenyl-1H-1,2,3-triazol-4-yl)-1-phenyl-1H-pyrazol-4-yl)methyleneamino)-1H-pyrazole-4-carbonitrile (**8**): Orange crystals from Dioxane in yield 91%, m.p. 161–163 °C. H^1^-NMR (400 MHz, DMSO-d_6_): δ = 2.53 (s, 3H, CH_3_), 6.32 (s, 1H, CH-pyrazole), 6.67 (s, 1H, CH-pyrazole), 7.35–7.84 (m, 10H, Ar-H), 8.50 (s, 1H, CH-aliphatic), 9.11 (s, 1H, NH). IR (KBr, cm^−1^): *v* 1600v (C=C), 1616 (C=N), 2222 (CN), 2858, 2974 (CH-aliphatic), 3105 (NH). MS: *m*/*z* (M^+^ 419, 21%), (M+1, 420, 15%), (M+2, 421, 4%). Anal. Calcd. for C_23_H_17_N_9_ (419): C, 65.86; H, 4.09; N, 30.05; Found: C, 65.92; H, 4.03; N, 30.01%.

N-((3-(5-methyl-1-phenyl-1H-1,2,3-triazol-4-yl)-1-phenyl-1H-pyrazol-4-yl)methylene)-3-phenyl-1H-pyrazol-5-amine (**9**): Beige crystals from Dioxane in yield 85%, m.p. 140–142 °C. H^1^-NMR (400 MHz, DMSO-d_6_): δ = 2.53 (s, 3H, CH_3_), 6.31 (s, 1H, CH-pyrazole), 6.68 (s, 1H, CH-pyrazole), 7.15–7.54 (m, 15H, Ar-H), 8.51 (s, 1H, CH-aliphatic), 9.11 (s, 1H, NH). IR (KBr, cm^−1^): *v* 1600 (C=C), 1616 (C=N), 2858, 2984 (CH-aliphatic), 3109 (NH). MS: *m*/*z* (M^+^, 470, 31%), (M+1, 471, 19%), (M+2, 472, 9%). Anal. Calcd. for C_28_H_22_N_8_ (470): C, 71.47; H, 4.71; N, 23.81; Found: C, 71.46; H, 4.72; N, 23.82%.

4-amino-1,6-dihydro-3-((3-(5-methyl-1-phenyl-1H-1,2,3-triazol-4-yl)-1-phenyl-1H-pyrazol-4-yl)methylene)-6-thioxopyridin-2(3H)-one (**10**): A mixture of compound number 1 (3.29, 10 mmol), ethylcyanoacetate (10 mmol) and thiourea (10 mmol) was heated under reflux in absolute ethanol (20 mL) containing a catalytic amount of hydrochloric acid (1 mL) for (15 h); the solid formed while heating was separated and recrystallized from dioxane as yellow crystals in 87% yield. m.p. 230–232 °C. H^1^-NMR (400 MHz, DMSO-d_6_): δ = 2.53 (s, 3H, CH_3_), 6.31 (s, 1H, CH-pyrazole), 6.82 (s, 2H, NH_2_), 7.5–7.8 (m, 10H, Ar-H), 8.21 (s, 1H, CH-aliphatic), 9.50 (s, 1H, NH). IR (KBr, cm^−1^): v 1400 (C=S), 1600 (C=C), 1620 (C=N), 1639 (C=O), 2852, 2984 (CH-aliphatic), 3228 (NH), 3317, 3348 (NH_2_). MS: *m*/*z* (M^+^, 454, 51%), (M+1, 455, 14%), (M+2, 456, 12%). Anal. Calcd. for C_23_H_18_N_8_OS (454): C, 60.78; H, 3.99; N, 24.65; Found: C, 60.76; H, 3.98; N, 24.64%.

1-(4-methoxyphenyl)-3-(3-(5-methyl-2-phenyl-2H-1,2,3-triazol-4-yl)-1-phenyl-1H-pyrazol-4-yl)prop-2-en-1-one (**11**): Sodium hydroxide (10 mL, 10%) was added dropwise to a mixture of compound number 3 (3.29, 10 mmol) and p-methoxy acetophenone (10 mmol) in ethanol (20 mL) while stirring at 0–5 °C. The stirring was continued for 2 h. The resulting solid was collected and recrystallized from ethanol to give compound number **11** as white crystals in 94% yield. m.p. 168–170 °C. H^1^-NMR (400 MHz, DMSO-d_6_): δ = 2.53 (s, 3H, CH_3_), 3.11 (s, 3H, OCH_3_), 6.51 (s, 1H, CH-pyrazole), 7.3–7.69 (m, 15H, Ar-H and CH=), 796–7.99 (d, 1H, J=12.0Hz, CH=). IR (KBr, cm^−1^): *v* 1662 (C=O), 2842, 2921 (CH-aliphatic). MS: *m*/*z* (M^+^, 461, 31%), (M+1, 462, 10%), (M+2, 463, 2%). Anal. Calcd. for C_28_H_23_N_5_O_2_ (461): C, 72.87; H, 5.02; N, 15.17; Found: C, 72.81; H, 5.06; N, 15.13%.

### 3.5. Synthesis of Compounds **13** and **14**

#### General Producer

Method **A**: a mixture of the compound **11** (10 mmol) and the appropriate amount of ethyl cyanoacetate or malononitrile (10 mmol) and ammonium acetate (0.77 gm, 10 mmol) was heated under reflux in acetic acid (20 mL) for 5 h. On cooling, the separated solid was filtrated, washed with water and recrystallized from the proper solvent to give the corresponding compounds 13 and 14 respectively in a good yield.

Method **B**: A mixture of the compound 1 (10 mmol), p-methoxy acetophenone (10 mmol), the appropriate amount of ethyl cyanoacetate or malononitrile (10 mmol), and an excess amount of ammonium acetate (4 gm) was heated under reflux in n-butanol (20 mL) for 3 h. On cooling, the separated solid was filtrated, washed with water and recrystallized from the proper solvent to give compounds identical in all aspects (m.p., IR, Mass, NMR) with that obtained from method **A**.

Ethyl 2-amino-6-(4-methoxyphenyl)-4-(3-(5-methyl-1-phenyl-1H-1,2,3-triazol-4-yl)-1-phenyl-1H-pyrazol-4-yl)pyridine-3-carboxylate (**13**): Yellow crystals from ethanol in yield 73%, m.p. 250–252 °C. H^1^-NMR (400 MHz, DMSO-d_6_): δ = 1.02–1.21 (t, 3H, CH_2_CH_3_), 2.53 (s, 3H, CH_3_), 3.11 (s, 3H, OCH_3_), 3.90–4.12 (q, 2H, CH_2_CH_3_), 6.34 (s, 1H, CH-pyrazole), 7.07–7.67 (m, 14H, Ar-H), 6.15 (s, 2H, NH_2_), 8.50 (s, 1H, CH-pyridine). IR (KBr, cm^−1^): *v* 1590 (C=C), 1601 (C=N), 1740 (C=O), 3066, 3133 (NH_2_). MS: *m*/*z* (M^+^, 571, 12%), (M+1, 572, 3%). Anal. Calcd. for C_33_H_29_N_7_O_3_ (571): C, 69.34; H, 5.11; N,17.15; Found: C, 69.28; H, 5.03; N, 17.10%.

2-Amino-6-(4-methoxyphenyl)-4-(3-(5-methyl-1-phenyl-1H-1,2,3-triazol-4-yl)-1-phenyl-1H-pyrazol-4-yl)pyridine-3-carbonitrile (**14**): Yellow crystals from ethanol in yield 71%, m.p. 271–273 °C. H^1^-NMR (400 MHz, DMSO-d_6_): δ = 2.53 (s, 3H, CH_3_), 3.11 (s, 3H, OCH_3_), 6.11 (s, 2H, NH_2_), 6.34 (s, 1H, CH-pyrazole), 7.37–8.10 (m, 14H, Ar-H), 8.50 (s, 1H, CH-pyridine). IR (KBr, cm^−1^): *v* 1592 (C=C), 1610 (C=N), 2212 (CN), 3350, 3417 (NH_2_). MS: *m*/*z* (M^+^, 524, 36%). Anal. Calcd. for C_31_H_24_N_8_O (524): C, 70.98; H, 4.16; N, 21.36; Found: C,70.91; H, 4.12; N, 21.25%.

4-(4-(4,5-dihydro-3-(4-methoxyphenyl)-1H-pyrazol-5-yl)-1-phenyl-1H-pyrazol-3-yl)-5-methyl-1-phenyl-1H-1,2,3-triazole (**17**): A mixture of compound number **11** (5 mmol) and hydrazine hydrate (1 gm, 1 mL, 10 mmol) was heated under reflux in ethanol (10 mL) for 3 h. The reaction mixture was cooled and the resulting solid collected and recrystallized from ethanol as white crystals in yield 76%. Yellow crystals from ethanol in yield 71%, m.p. 291–293 °C. H^1^-NMR (400 MHz, DMSO-d_6_): δ = 2.53 (s, 3H, CH_3_), 3.11 (s, 3H, OCH_3_), 3.44 (dd, 1H, *J* = 18.1, 5.8Hz, CH_2_-pyrazole), 3.89 (dd, 1H, *J* = 18.1, 12Hz, CH_2_-pyrazole), 4.99 (dd, 1H, *J* = 12.2,5.8, CH-pyrazole), 6.34 (s, 1H, CH-pyrazole), 7.07–7.66 (m, 15H, Ar-H and NH). IR (KBr, cm^−1^): *v* 1597 (C=C), 1620 (C=N), 3311 (NH). MS: *m*/*z* (M^+^475, 22%), (M+2477, 14%). Anal. Calcd. for C_28_H_25_N_7_O (475): C,70.72; H, 5.30; N, 20.62; Found: C,70.72; H,5.29; N,20.61%.

4,5-Dihydro-3-(4-methoxyphenyl)-5-(3-(5-methyl-1-phenyl-1H-1,2,3-triazol-4-yl)-1-phenyl-1H-pyrazol-4-yl)pyrazole-1-carbothioamide (**20**): A mixture of 1-(4-methoxyphenyl)-3-(3-(5-methyl-2-phenyl-2H-1,2,3-triazol-4-yl)-1-phenyl-1H-pyrazol-4-yl)prop-2-en-1-one 11. (2.5 g, 5 mmol), thiosemicarbazide (0.46 g, 5 mmol) in ethanol (20 mL) containing a catalytic amount of hydrochloric acid (0.5 mL) was heated under reflux for 3 h. The resulting solid was collected and recrystallized from dioxane to give 20 as white crystals in Yield: 82%, m.p. 207–209 °C. H^1^-NMR (400 MHz, DMSO-d_6_): δ = 2.54 (s, 3H, CH_3_), 3.11 (s, 3H, OCH_3_) 3.40–3.47 (dd, 1H, *J* = 18.1, 5.8Hz, CH_2_-pyrazole), 3.74–3.82 (m, 2H, CH_2_-pyrazole), 4.61 (s, broad, 2H, NH_2_), 7.01–7.72 (m, 15H, Ar-H, CH-pyrazole). IR (KBr, cm^−1^): *v* 3475, 3436 (NH_2_), 2924, 2890 (CH-alphatic), 1562 (C=C), 1616 (C=N). MS: *m*/*z* (M^+^534, 96%), (M^+1^ 535, 3%), (M^+2^ 536, 11%). Anal. Calcd. for C29H_26_N_8_OS (534): C, 65.15; H, 4.90; N, 20.69; Found: C, 65.14; H, 4.91; N, 20.69%.

## 4. Biological Experiments

### 4.1. α-Reductase Inhibitors

#### 4.1.1. Treatment of Animals

Animals were obtained from the animal house colony of the NRC Cairo Egypt. All animals were allowed free access to water and were kept on a constant standard diet. Nineteen Groups, each of 12 male Sprague–Dawley rats in the postnatal 3rd day were treated subcutaneously with the 5α-reductase inhibitor (tested compound or reference standard). The tested compounds were suspended in 5% Tween 80 in water. The vehicle was used for both the standard and negative control group, beginning on the postnatal 3rd day until the age of 7 weeks. Eighteen Groups were used to test the activities, of which one was used as the positive control for anastrozole and another served as the negative control group. After scarifying, blood was withdrawn for testosterone and dihydrotestosterone (DHT) determination [34]. Moreover, intraprostatic concentrations of testosterone and DHT were determined [35]. The biological experiments were performed according to the official standards.

#### 4.1.2. Aromatase Activity Assay

Inhibitory potencies of compounds were determined according to an established procedure using a commercially available aromatase test kit from BD Gentest [36]. This fluorescence-based assay measures the rate at which recombinant human aromatase (baculovirus/insect cell-expressed) converts the substrate 7-methoxy-trifluoromethylcoumarin (MFC) into a fluorescent product 7-ethynyl-trifluoromethylcoumarin (HFC) (λ_ex_ = 409 nm, λ_em_ = 530 nm) in a NADPH-regenerating system. Briefly, concentrated stock solutions of test compounds were prepared in acetonitrile.

One-hundred microliter samples containing serial dilutions of test compounds (dilution factor of 3 between samples) and cofactor mixture (0.4 U/mL glucose-6-phosphate dehydrogenase; 16.2 µM NADP^+^; 825 µM MgCl_2_; 825 µM glucose -6-phosphate; 50 µM citrate buffer, pH 7.5) were prepared in a 96-well plate. After incubating the plate for 10 min at 37 °C, 100 µL of an aromatase/P450 reductase/substrate solution (105 µg protein/mL enzyme; 50 µM MFC; 20 mM phosphate buffer, pH 7.4) were added to each well. The plate was covered and incubated for 30 min at 37 °C. Seventy-five microliters of 0.5 M Tris base were then added to stop the reaction, and the fluorescence of the formed de-methylated MFC was measured with a plate reader (SpectraMax Gemini, Molecular Devices).

Fluorescence intensities, which were proportional to the amount of reaction product generated by aromatase, were graphed as a function of inhibitor concentration and then fit to a three-parameter logistic function. Inhibitory potencies were expressed in terms of the IC_50_ value, the inhibitor concentration necessary to reduce the enzyme activity by half. Each experiment was performed at least in triplicate.

## 5. Conclusions

Results of the present study demonstrate that compound **1** reacts with different active methylene and amino pyrazoles to yield a new series of pyrazole derivatives derived from 1,2,3-triazole derivative. Newly synthesized compounds **1**–**11**, **13**, **14**, **17,** and **20** were evaluated in vivo as 5α-reductase and aromatase inhibitors; most of the tested compounds exhibited more potent activity than the standard references drugs. Compound **13** has the highest potency amongst the tested compounds and the reference drugs, and this may be due to the presence of the pyridine moiety linked with pyrazoline and 1,2,3-trizole moieties. The combination between 1,2,3-triazole and pyrazole was found to be essential for 5α-reductase and aromatase inhibitor activities. Future work will involve the design of steroidal molecules of such features.

## Figures and Tables

**Table 1:** 5α-Reductase inhibitors activities and acute toxicity of the studied compounds.

Compound	IC_50_ (µM)
1	0.00761
2	0.00454
3	0.00343
4	0.00122
5	0.00213
6	0.00192
7	0.00091
8	0.00021
9	0.00192
10	0.00171
11	0.00026
13	0.00015
14	0.00074
17	0.00063
20	0.00053
Anastrozole	0.00241

**Table 2:** Aromatase inhibitors’ activities of compounds **1–14**, **17** and **20**.

Compound	IC_50_ (µM)
1	0.00913
2	0.00724
3	0.00635
4	0.00566
5	0.00454
6	0.00342
7	0.00253
8	0.00144
9	0.00233
10	0.00142
11	0.00033
13	0.00024
14	0.00093
17	0.00084
20	0.00064
Letrozole	0.00280
